# Comparative Study of Functional Outcomes Between Cemented and Uncemented Total Hip Replacement at a Tertiary Care Hospital in Odisha, India

**DOI:** 10.7759/cureus.85444

**Published:** 2025-06-05

**Authors:** Aniruddh Dash, Sourav Mishra, Spandan Mishra, Nihar R Mishra, Sunit Pani, Chaitanya Khandelwal, Abhinav Sharma, Shivam Chawla

**Affiliations:** 1 Department of Orthopaedics, Institute of Medical Sciences and SUM Hospital, Bhubaneswar, IND; 2 Orthopaedics and Trauma, Institute of Medical Sciences and SUM Hospital, Bhubaneswar, IND

**Keywords:** cemented vs uncemented, eastern odisha, harris hip score, total hip replacement (thr), vas for pain, womac

## Abstract

Background

This study aims to assess the clinical and functional outcomes of cemented and uncemented total hip replacement (THR) by comparing the two surgical techniques in Odisha. Also, it aims to assess complications of THR/total hip arthroplasty (THA) by comparing two surgical techniques as well.

Materials and methods

This prospective observational study was conducted in the Department of Orthopedics and Trauma, Institute of Medical Sciences and SUM Hospital, Bhubaneswar, from March 2022 to September 2023. A total of 60 patients undergoing primary THR were enrolled and categorized into cemented and uncemented groups. Clinical evaluations were conducted using the Visual Analogue Scale (VAS), Harris Hip Score (HHS), and Western Ontario and McMaster Universities Osteoarthritis Index (WOMAC) at six weeks, three months, six months, and 12 months.

Results

The mean age of patients in the cemented THR group was 55.53 years, while the uncemented group had a mean age of 52.60 years. Pain levels, assessed using the VAS, were similar between groups at the six-week follow-up. However, at three months (p ≤ 0.0001) and six months (p = 0.0001), the cemented group reported significantly lower VAS scores, indicating more effective early pain control and likely improved initial osseointegration. Functional outcomes, measured by the HHS and the WOMAC, also favored the cemented group. Statistically significant differences were observed at three months (p = 0.001), six months (p ≤ 0.05), and at the one-year follow-up (p ≤ 0.05). There was one case of excessive blood loss during surgery in the uncemented group and one case of foot drop in the cemented group. Sixty-six patients were included, but six left the study - four did not meet the inclusion criteria, and two declined to provide consent.

Conclusion

Cemented implants are a more cost-effective option, offering improved short-term outcomes, and also allow for full weight-bearing activities with minimal discomfort. Early movement and mobilization are seen in cemented implants, while uncemented implants offer better durability and avoid cement-related complications, with stronger biological integration. Although the decision between cemented and uncemented THR depends on various patient-specific factors, including age, activity level, bone quality, and overall health, each method has its own risks and benefits that should be considered after detailed deliberation.

## Introduction

Total hip replacement (THR) is an established and accepted orthopaedic surgery for the treatment of advanced hip diseases. Patients with hip diseases are disabled because of pain, instability, decreased range of motion, and loss of gait function. The goal of arthroplasty is to produce a pain-free, functional, freely moving, and long-lasting joint. Success rates of 80-85% and more have been reported since Charnley pioneered his arthroplasty procedure using polymethyl methacrylate cement to attain fixation of femoral and acetabular components [1]. Early hip evaluation research by D'Aubigné and Postel, and by Charnley, followed post-operative arthroplastic outcomes by evaluating pain, range of motion, and functional status of the patients [2]. Loosening rates between 10-50% on the femoral side and 5-20% on the acetabular side stimulated medical research into alternatives to cemented fixation [3]. Among the various methods that have been investigated as alternatives to polymethyl methacrylate cement fixation, biologic fixation of prosthesis with porous coatings had the most promising results in terms of relief of pain, improved activity level, range of movement and walking efficiency, and radiological assessment of loosening. These concerns led to the development of cementless implants utilizing porous coatings that promote osseointegration and provide long-term stability without cement [4].

Rehabilitation of patients with cemented and uncemented THRs follows similar postoperative protocols except for the weight-bearing status of the patient. There are currently multiple opinions regarding the weight-bearing status of patients after undergoing the uncemented procedure, as one study shows no evidence that late weight-bearing after uncemented total hip arthroplasty (THA) implies any serious adverse effects on functional recovery after 24 weeks compared with immediate postoperative weight-bearing. Immediate unrestricted weight bearing (UWB) did not have extra harm compared with partial weight bearing (PWB) in patients undergoing uncemented THA. UWB was not superior to PWB. Considering the improvement of the Harris Hip Score (HHS) score and the compliance of patients, UWB can be encouraged in THA rehabilitation.

A THR is a highly successful and affordable procedure designed to relieve joint pain and help you move more freely. Whether it’s walking, climbing stairs, or simply enjoying your daily routine, this surgery can help you get back to doing the things you love with greater comfort and confidence. Since THRs were first introduced, advances in technology have continuously improved outcomes, leading to better joint function and longer-lasting implants. There are two main types of implant fixation: cemented and cementless. Cemented implants are secured using a special bone cement that locks the implant in place once it sets. Cementless implants, on the other hand, are designed to fit tightly into the bone and rely on the body’s natural healing process, allowing new bone to grow onto or into the implant over time for long-term stability. Technological advancements have improved THR outcomes regardless of fixation type [5,6].

There have been massive advancements in the field of THR, but still the very debate: cemented or uncemented? Despite the significant strides made in implant design, materials, and surgical techniques, the debate surrounding the use of cement in THRs persists. While some argue that cemented implants provide greater initial stability, others contend that uncemented options offer superior long-term outcomes. As researchers and clinicians, it is essential that we continue to investigate and refine our understanding of this fundamental issue, ultimately striving to provide the most effective outcomes. At our institute, as well as other institutes, uncemented THRs have been reported to account for more than 95% of cases [7]. There is an advantage of uncemented THR in young patients as they require revision. However, in older patients who are older than 50 years, cemented THR, being cheaper, can be a better option if there is no difference in functional outcome.

## Materials and methods

This prospective observational study was conducted in the Department of Orthopedics and Trauma, Institute of Medical Sciences and SUM Hospital, Bhubaneswar, over a period of one year and six months. A convenient sampling technique was employed to achieve the desired sample size. A total of 60 patients were enrolled and divided into two groups: Group A consisted of patients who underwent cemented THR, and Group B included patients who underwent uncemented (non-cemented) THR.

Prior to the initiation of the study, approval was obtained from the Institutional Ethics Committee (IEC/IMS.SH/SOA/2024/680). Patients who met the predetermined inclusion and exclusion criteria and provided written informed consent were included. A minimum of 60 cases with clear clinical indications for THR were selected.

The inclusion criteria comprised male and female patients aged between 30 and 75 years, who were medically fit for surgery and had provided informed consent. Patients included had specific indications for THR, such as grade 3 or grade 4 avascular necrosis (AVN). Patients were excluded if they were medically unstable or unfit for surgery, unwilling to undergo surgery, or had osteoporotic or pathological fractures. Additional exclusion criteria included recent dental procedures, compound injuries, previous surgical interventions, pregnancy, immunosuppression, Dorr type C femoral morphology, neurovascular deficits, active infections, psychiatric disorders, and those with grade 1 or grade 2 AVN.

Data collection and patient evaluation were based on several clinical parameters. A detailed history was taken, including the mode of injury, duration of symptoms, and any previous treatment received. Clinical examination was performed systematically, with a focus on local and neurovascular assessment. Radiological evaluation included standard X-rays and computed tomography (CT) scans; additional imaging modalities were used when necessary. Baseline and relevant investigations were also conducted. The diagnosis was established through a combination of clinical and radiological findings.

The surgical procedure involved THR performed using the Hardinge approach. All procedures were conducted by a single surgeon to maintain consistency. Indications for surgery included osteoarthritis, rheumatoid arthritis, or other inflammatory arthropathies, post-traumatic degenerative joint disease, and osteonecrosis or joint collapse with cartilage destruction. Routine antibiotic prophylaxis and analgesic or anti-inflammatory medications were administered perioperatively.

Postoperative evaluation involved both clinical examination and radiological assessment using X-ray. Complications were assessed at three stages: preoperative, immediate postoperative, and late postoperative periods.

## Results

**Figure 1:**
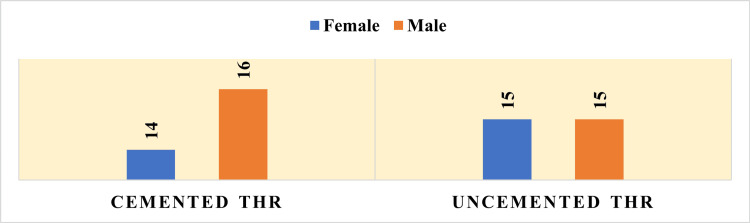
Gender distribution in each group The cemented total hip replacement (THR) group consisted of 14 females and 16 males, while the uncemented THR group included 15 females and 15 males.

The Chi-square test indicated no significant difference in gender distribution between the two patient groups, with a p-value of 0.50 (p > 0.05). Consequently, the relationship between age, sex, and duration of disease across the two groups was found to be statistically insignificant in influencing the study outcomes, as shown in Figure 1.

Among the 30 cases in the cemented group, four (13.33%) were diagnosed with arthritis, 10 (33.33%) presented with AVN of the femoral head, one (3.33%) had a history of hip dislocation, eight (26.66%) sustained a fracture of the femoral neck, three (10%) demonstrated non-union of intertrochanteric femoral fractures, two (6.66%) had non-union of the femoral neck, one (3.33%) was diagnosed with rheumatoid arthritis of the hip, and one (3.33%) was affected by tuberculous involvement of the hip.

**Figure 2:**
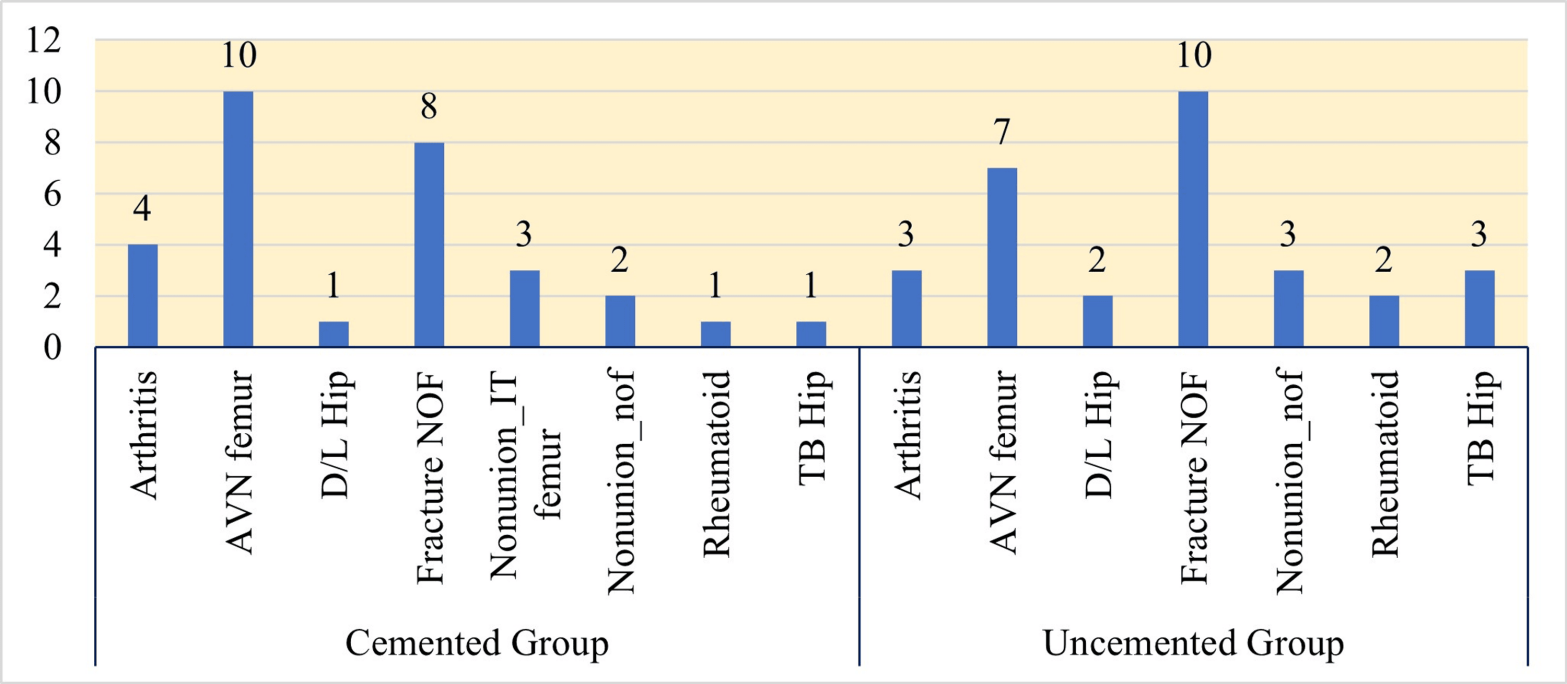
Distribution of diagnoses among patients in each group AVN: avascular necrosis; D/L hip: dislocated hip; NOF: neck of femur; nonunion IT femur: nonunion intertrochanteric fracture of the femur; TB hip: tuberculous hip

Among the 30 cases in the uncemented group, one (3.33%) was diagnosed with arthritis, seven (23.33%) presented with AVN of the femoral head, two (6.66%) had a history of hip dislocation, 10 (33.33%) sustained fractures of the femoral neck, three (10%) had non-union of the femoral neck, two (6.66%) were diagnosed with rheumatoid arthritis, and three (10%) had tuberculous involvement of the hip. THR was performed for all these conditions, as depicted in Figure 2.

A comparison between cemented (Group A) and uncemented (Group B) THR, as shown in Table 1, showed no significant difference in patient age. In terms of outcomes, the Western Ontario and McMaster Universities Osteoarthritis Index (WOMAC) B scores, which assess joint stiffness, were significantly better in the uncemented group. WOMAC A scores, which measure pain and physical function, were comparable at six weeks but significantly improved in Group B at three months, six months, and one year postoperatively. These findings suggest that uncemented THR offers superior long-term pain relief and functional recovery, along with reduced stiffness.

**Table 1:** Comparison of age (in years) and WOMAC scores in both groups Values are presented as mean ± standard deviation. P-values were calculated using the independent t-test. For comparisons between the cemented and uncemented THA groups (n=30 each, df = 58), the critical t-value at α = 0.05 (two-tailed) is approximately 2.000; any calculated t-statistic exceeding this indicates a statistically significant difference. WOMAC: Western Ontario and McMaster Universities Osteoarthritis Index; SD: standard deviation; n: number of participants; THA: total hip arthroplasty

Variables	Group A (n=30)	Group B (n=30)	P-value
Age			
Mean ± SD	55.53 ± 11.41	52.60 ± 13.61	0.115
WOMAC B Scores			
Mean ± SD	81.50 ± 2.54	87.00 ± 1.01	0.0001
WOMAC A at 6 weeks			
Mean ± SD	53.50 ± 0.50	59.50 ± 0.50	1.00
WOMAC A at 3 months			
Mean ± SD	48.50 ± 0.50	56.00 ± 1.01	0.0001
WOMAC A at 6 months			
Mean ± SD	48.00 ± 2.03	43.00 ± 1.01	0.0001
WOMAC A at 1 year			
Mean ± SD	46.60 ± 2.11	40.00 ± 1.12	0.0001

Table 2 presents a comparison of HHS between the cemented and uncemented groups. Although early postoperative HHS scores at six weeks were similar between cemented and uncemented THR groups, uncemented THR showed significantly better overall functional outcomes. Group B (uncemented) had higher baseline (Harris B) scores and demonstrated superior long-term recovery at six months and one year. These findings suggest better sustained hip function and mobility with uncemented THR.

**Table 2:** Comparison of Harris Hip Scores in cemented THR group and uncemented THR group Harris B score reflects preoperative hip function, while Harris A score measures postoperative recovery at various follow-up intervals. Values are expressed as mean ± standard deviation. P-values were calculated using the Independent t-test. For the Harris score comparisons (n=30 per group, df=58), the critical t-value at α = 0.05 (two-tailed) is approximately 2.000, indicating significance if the t-statistic exceeds this value. THR: total hip replacement; SD: standard deviation; n: number of participants

Variables	Group A (n=30)	Group B (n=30)	P-value
Harris B Scores			
Mean ± SD	84.50 ± 1.52	90.50 ± 0.50	0.0001
Harris A at 6 weeks			
Mean ± SD	56.00 ± 1.01	56.50 ± 1.52	0.14
Harris A at 3 months			
Mean ± SD	50.50 ± 0.50	49.00 ± 1.01	0.0001
Harris A at 6 months			
Mean ± SD	47.50 ± 0.44	41.50 ± 2.54	0.0001
Harris A at 1 year			
Mean ± SD	46.50 ± 20.50	35.00 ± 2.03	0.0001

Table 3 presents a comparison of Visual Analogue Scale (VAS) scores between the cemented (Group A) and uncemented (Group B) THR groups. Both cemented (Group A) and uncemented (Group B) THR groups showed significant pain reduction over time. However, Group B (uncemented) demonstrated better long-term pain relief, with significantly lower VAS scores at six months and one year compared to Group A (cemented), suggesting superior pain management in the uncemented group.

**Table 3:** Comparison of VAS scores in cemented THR group and uncemented THR group VAS B measures preoperative pain, while VAS A assesses postoperative pain at different follow-up intervals. Values are expressed as mean ± standard deviation. P-values were calculated using the Independent t-test. For the VAS score comparisons (n=30 per group, df = 58), the critical t-value at α = 0.05 (two-tailed) is approximately 2.000, indicating statistical significance if the t-statistic exceeds this value. VAS: Visual Analog Scale; SD: standard deviation; n: number of participants; THR: total hip replacement

Variables	Group A (n=30)	Group B (n=30)	P-value
VAS B Scores			
Mean ± SD	8.50 ± 0.50	9.00 ± 0.00	0.0001
VAS A at 6 weeks			
Mean ± SD	7.50 ± 0.50	7.50 ± 1.52	0.0001
VAS A at 3 months			
Mean ± SD	4.50 ± 0.50	6.00 ± 2.03	0.0001
VAS A at 6 months			
Mean ± SD	4.50 ± 0.50	3.50 ± 0.50	0.0001
VAS A at 1 year			
Mean ± SD	3.00 ± 0.00	2.00 ± 0.00	0.0001

During the surgical and postoperative phases for both groups undergoing THR, a few complications arose. In the uncemented group, there was one incident of significant blood loss during the procedure, while the cemented group experienced a case of foot drop, affecting 2% of the patients. In this particular case, the patient's sensation remained intact, and plantar flexion was preserved. The occurrence of foot drop was likely due to neuropraxia resulting from improper retraction during surgery. Conservative treatment of foot drop included ankle-foot orthosis, oral medications, physical therapy, functional electrical stimulation, and activity modification. The patient achieved a complete recovery within approximately three months. All surgeries were performed using the Hardinge technique, also known as the direct lateral approach, by a single surgeon.

## Discussion

THR surgery has made significant strides and is a highly effective option for patients suffering from hip joint damage. Over the years, various innovative designs have emerged, each claiming to offer distinct advantages over the others. However, these advancements have also led to increased costs for patients. A fundamental question persists: Should cement be used or not?

This question is particularly pressing for elderly patients and in developing countries like India, where cost-effectiveness remains a critical issue. In the last decade, there has been a noticeable global shift toward uncemented THR, introduced to address complications associated with cemented procedures, particularly in younger patients. Nevertheless, many institutions have now shifted their focus towards performing cementless THRs. We excluded the Dorr type C femur to make it a non-biased study. We compared cemented and uncemented THR in terms of functional VAS score, HHS, and WOMAC outcome.

Our study found that during the first six weeks of follow-up, patients who underwent both cemented and uncemented THR reported similar levels of pain in the affected area. While those in the cemented group exhibited slightly higher pain scores compared to the uncemented group, over the course of one year, it became evident that patients in the cemented group demonstrated better compliance than those in the uncemented group. Previous literature corroborates these findings, particularly in older populations where cemented fixation offers enhanced initial stability [1,8].

Our study supports a similar approach, with cemented implants demonstrating significant advantages in early recovery and cost-effectiveness. Other studies also showed that cemented hips worked particularly well in patients who are obese or have weaker bones, like those with osteoporosis, and they also had fewer complications during surgery, such as fractures of the thigh bone.

Contrasting these findings, a meta-analysis by Morshed et al. [9] found no significant differences in the survival rates of the two types of implants. Meanwhile, a study published by Pennington et al. [10] stated data that showed fully uncemented hip replacements are more expensive, but over the first 12 months after surgery, there was no meaningful difference in clinical or functional outcomes compared to cemented ones. So, despite the higher cost, the results during that first year were essentially the same. Cementless THRs, although preferable in younger patients for potential revision ease, require prolonged rehabilitation and carry a risk of early migration [11,12]. Registry data from Sweden and other Nordic countries highlight a trend toward cementless fixation in younger demographics and cemented fixation in older adults, driven by bone quality and economic considerations [13,14].

**Figure 3:**
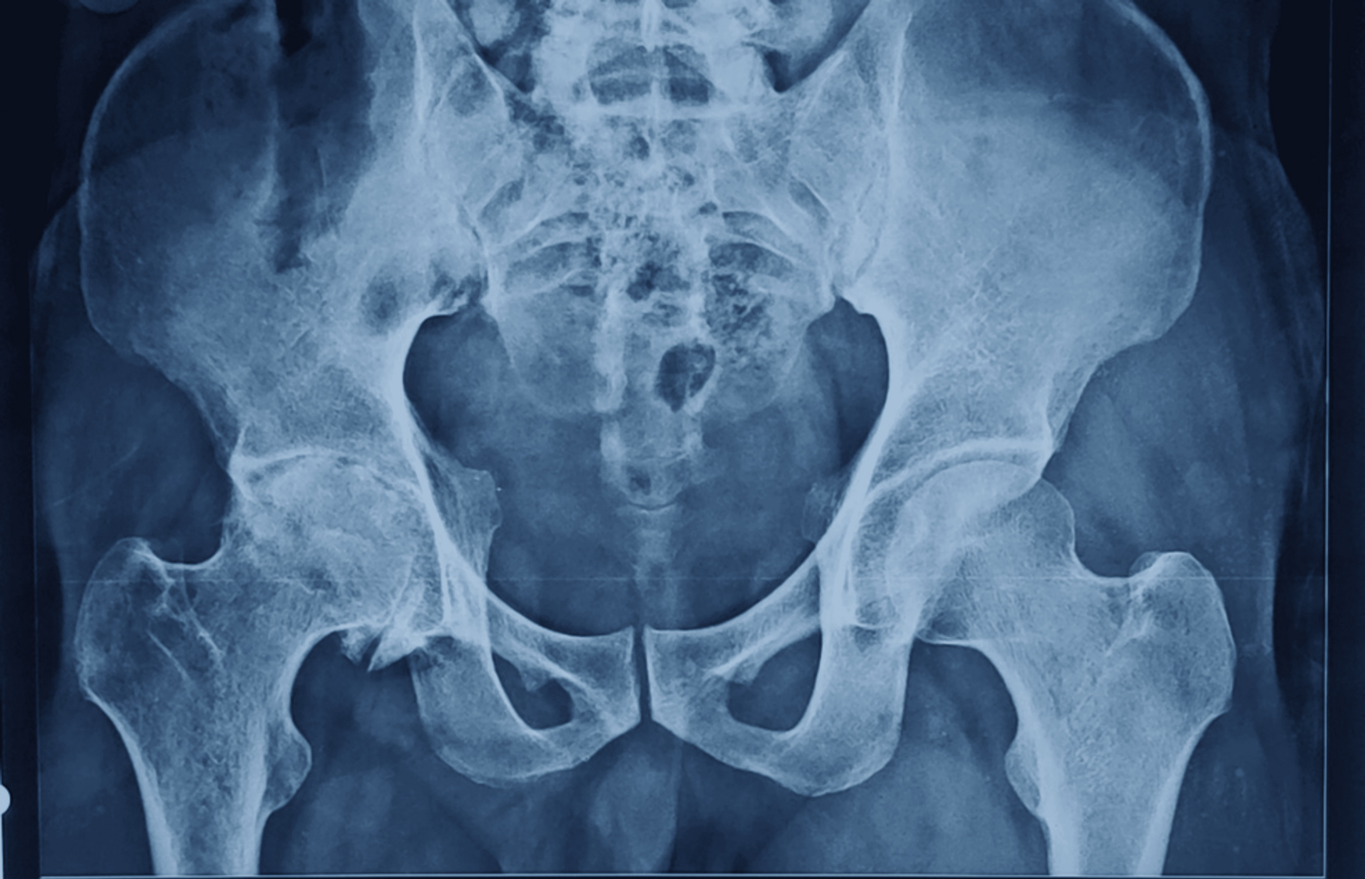
Case 1: Pre-operative X-ray of a 34-year-old male showing AVN of the right hip. AVN: avascular necrosis

**Figure 4:**
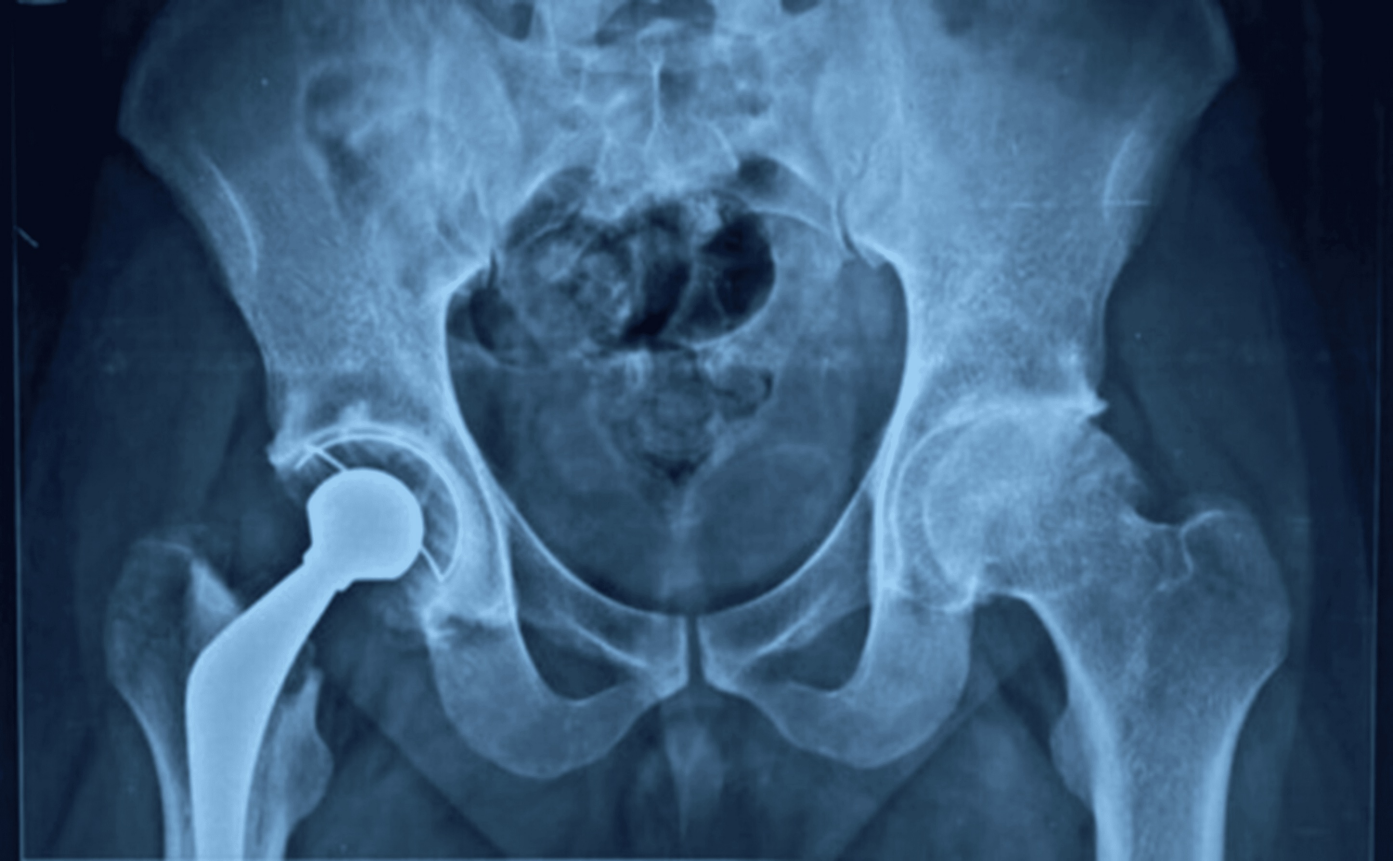
Case 1: Post-operative X-ray showing cemented THR of the right hip. THR: total hip replacement

**Figure 5:**
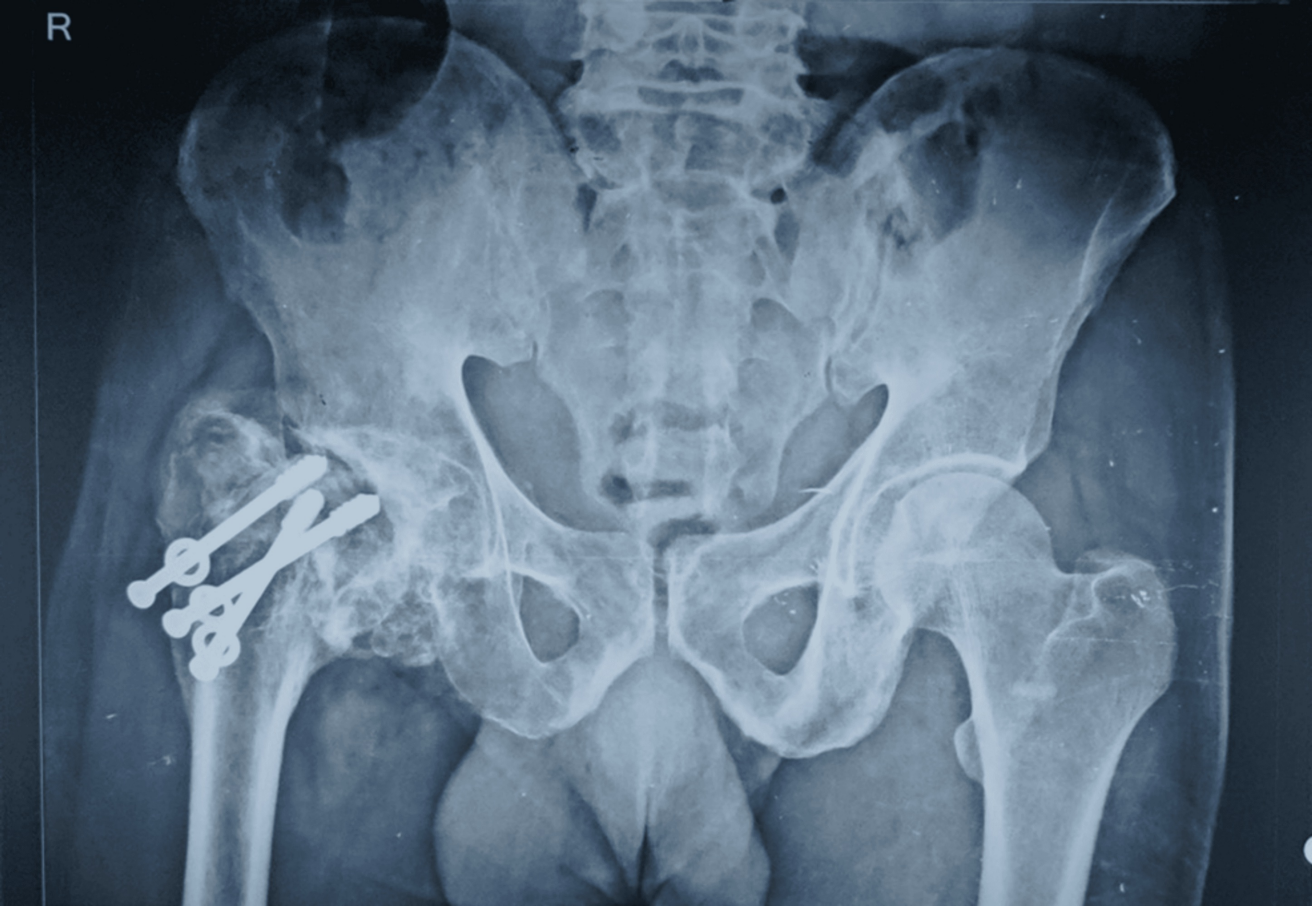
Case 2: Pre-operative X-ray of a 56-year-old male with secondary osteoarthritis of the right hip and implant in situ.

**Figure 6:**
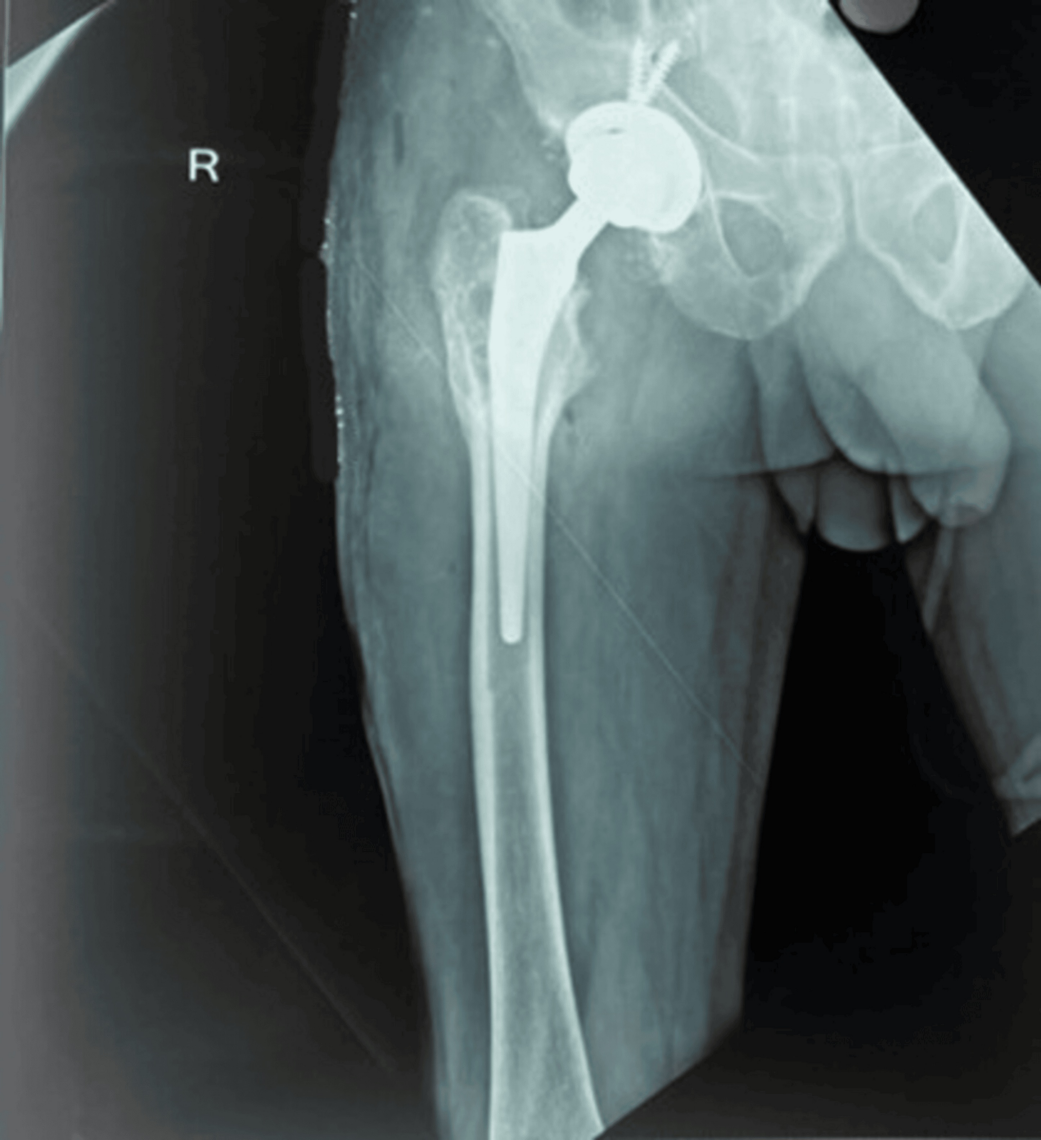
Case 2: Post-operative X-ray showing uncemented THR of the right hip. THR: total hip replacement

The short-term clinical outcomes indicated satisfactory pain relief and early mobilization. For patients in economically challenged settings, particularly those over 50-55 years of age, cemented THR prostheses may represent a viable treatment choice due to their affordability [13]. It is important to recognize that major complications associated with THA, like aseptic loosening and periprosthetic osteolysis, often manifest later, indicating that a one-year follow-up may be insufficient. Therefore, further longitudinal studies are essential to fully assess the long-term outcomes associated with these implants. Data regarding reoperation rates and the underlying causes after THRs highlights a significant issue: the swiftly increasing demand for revision THRs and the challenges that come with it. This trend poses a serious threat to clinical care quality and places additional financial burdens on healthcare systems. Therefore, it is crucial for policymakers to implement timely interventions and to formulate effective healthcare strategies to address these concerns. Figures 3-4 depict the pre-operative and post-operative X-rays of a patient who underwent cemented THR. Figures 5-6 depict the pre-operative and post-operative X-rays of a patient who underwent uncemented THR.

The study's limitations include a small sample size, a non-randomized design, and being conducted at a single center, which may limit generalizability. A short follow-up duration and reliance on subjective outcome measures could affect the robustness of the findings. Additionally, strict inclusion and exclusion criteria, potential observer bias, and the lack of a control group or complication assessment are notable constraints.

## Conclusions

In conclusion, our study indicates cemented implants are generally more cost-effective and are associated with improved short-term outcomes, including the ability to resume full weight-bearing activities with minimal discomfort. They also facilitate early postoperative mobilization, which can be advantageous in certain patient populations. In contrast, uncemented implants offer superior long-term durability and eliminate the risk of cement-related complications, owing to their reliance on biological fixation and osseointegration for implant stability.

The choice between cemented and uncemented THR should be individualized, taking into account a range of patient-specific factors such as age, activity level, bone quality, and overall health status. Each approach presents distinct advantages and potential risks, and the decision should be made following a comprehensive clinical assessment and shared decision-making process.
