# Correction: Comparative Study of Functional Outcomes Between Cemented and Uncemented Total Hip Replacement at a Tertiary Care Hospital in Odisha, India

**DOI:** 10.7759/cureus.c433

**Published:** 2026-05-18

**Authors:** Aniruddh Dash, Sourav Mishra, Spandan Mishra, Nihar R Mishra, Sunit Pani, Chaitanya Khandelwal, Abhinav Sharma, Shivam Chawla

**Affiliations:** 1 Department of Orthopaedics, Institute of Medical Sciences and SUM Hospital, Bhubaneswar, IND; 2 Orthopaedics and Trauma, Institute of Medical Sciences and SUM Hospital, Bhubaneswar, IND

This article has been corrected to include Aniruddh Dash as first author after Dr. Dash was erroneously omitted from the submitted author list. All authors have provided written consent for the addition of Dr. Dash to the author list.

